# Age‐ and alcohol‐related differences in adolescent neurometabolite levels

**DOI:** 10.1111/acer.70256

**Published:** 2026-02-15

**Authors:** Maria I. Perica, Anna E. Kirkland, Louise Mewton, Lindsay M. Squeglia

**Affiliations:** ^1^ Department of Psychiatry and Behavioral Sciences Medical University of South Carolina Charleston South Carolina USA; ^2^ Matilda Centre for Research in Mental Health and Substance Use University of Sydney Sydney New South Wales Australia

**Keywords:** adolescence, alcohol, magnetic resonance spectroscopy, neurometabolite, neurotransmitter

## Abstract

**Background:**

Adolescence is a critical period for neurometabolite maturation as well as the initiation of alcohol use. However, despite its importance for long‐term neurodevelopmental outcomes, the impact of alcohol use during adolescence on neurometabolites remains understudied.

**Methods:**

We conducted an exploratory study using 3T proton magnetic resonance spectroscopy (1H‐MRS) to examine age‐ and alcohol‐related associations with six neurometabolites in the dorsal anterior cingulate cortex (dACC) that are involved in key neural functions: glutamate + glutamine (Glx), GABA plus macromolecules (GABA+), total N‐acetylaspartate (tNAA), total choline (tCho), total creatine (tCr), and *myo*‐inositol (mI). Participants were 84 adolescents (ages 17–22; 67% female) with moderate‐to‐high alcohol use who completed 1H‐MRS scans and self‐reported their alcohol use. Alcohol use variables included as follows: estimated lifetime drinking episodes and past 60‐day total drinking days, total binge drinking days, total number of drinks, and drinks per drinking day.

**Results:**

Older adolescents had higher levels of GABA+, tNAA, tCho, and mI, and lower levels of Glx and Glx/GABA+; tCr was not associated with age. More alcohol use—specifically more drinking days, binge drinking days, and number of drinks—was associated with lower tNAA levels. There were no associations with lifetime drinking episodes.

**Conclusions:**

In a sample of moderate‐to‐heavy drinking adolescents, findings suggest associations between age and neurometabolites in dACC neurometabolites, which potentially reflect ongoing neuronal maturation, myelination, and shifts in excitatory and inhibitory neurotransmission. Lower tNAA among recent heavier drinkers may reflect associations between alcohol exposure and neuronal damage. Broader neurometabolic effects may emerge only with heavier or prolonged alcohol use.

## Graphical abstract

Adolescence is a critical period for neurometabolic maturation and is typically when alcohol use is initiated. Using 3T magnetic resonance spectroscopy in 84 adolescents (ages = 17–22), we observed age‐related increases in metabolites supporting neuronal health, membrane synthesis, glial function, inhibitory neurotransmission, and decreases in excitatory neurotransmission. More alcohol use was associated with lower levels of a metabolite linked to neuronal health. These findings clarify developmental patterns of neurometabolites and highlight a potential biomarker for early alcohol‐related disruption during adolescence.

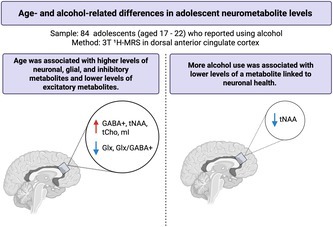

## INTRODUCTION

Adolescence is a significant and distinct period of brain development, marked by dynamic neurobiological changes in regions critical for higher‐order functions, such as cognition (Luna et al., 2015). Key neurodevelopmental processes during adolescence include thinning of cortical gray matter (Gogtay et al., 2004), pruning of synapses (Petanjek et al., 2011), myelination of axons (Baum et al., 2022), and changes in neurotransmitter systems (Larsen & Luna, 2018; Perica et al., 2022). However, adolescence is also a time when heightened activity in neural reward systems can lead to increased risk‐taking and sensation‐seeking behaviors, such as alcohol use (Spear, 2000). In fact, adolescence is often when alcohol use is first initiated and begins to escalate notably (Hingson et al., 2006a). This presents a significant public health concern, as earlier initiation of alcohol use has been linked to more severe long‐term consequences, including an increased risk of later meeting criteria for alcohol use disorder (AUD) (Dawson et al., 2008; Hingson et al., 2006a, 2006b). Moreover, alcohol use has been shown to significantly affect neurobiology, including neural processes that are actively developing during adolescence, which may lead to long‐term, negative consequences for neurodevelopmental outcomes (Hiller‐Sturmhöfel & Spear, 2018; Spear, 2018).

Despite these concerns, knowledge about how alcohol affects neurometabolite development in the human brain is limited. These neurometabolites have been linked to key neural functions (e.g., involvement in neurotransmitter systems and critical metabolic pathways) and include N‐acetylaspartate (NAA or total NAA [tNAA]; a marker for neuronal health, integrity, density), total choline‐containing metabolites (tCho; a putative marker for lipid membrane synthesis/turnover), GABA or GABA plus macromolecules (GABA+; an inhibitory neurotransmitter), *myo*‐inositol (mI; a putative marker for lipid membranes and glia), glutamate + glutamine (Glx; excitatory neurotransmitter glutamate + glutamine), and total creatine‐containing metabolites (tCr; a marker for energetic/metabolic activity) (Rae, 2014). Much of the known research thus far has been conducted in animal models and has identified potential widespread neurometabolite alterations as a result of alcohol binging, withdrawal, and craving (Koob, 2024). Additionally, a recent meta‐analysis of 43 proton magnetic resonance spectroscopy (1H‐MRS) studies investigating neurometabolite alterations as a result of alcohol use found that the human literature also notes widespread neurometabolic impacts; however, the studies were disproportionately based on samples of middle‐aged men, with no study samples under the average age of 21 (Kirkland et al., 2022). The overrepresentation of older, male samples limits our understanding of alcohol's impact across sexes during adolescence—a key developmental period of potential neurometabolic development (Ghisleni et al., 2015; Gleich et al., 2015; Perica et al., 2022; Shimizu et al., 2017; Silveri et al., 2013; Widegren et al., 2024). Given the unique neurodevelopmental processes occurring during human adolescence, findings from adult samples or animal models may not generalize to the adolescent brain. Indeed, age has been identified as a crucial factor influencing the efficacy and safety of psychopharmacological treatments for psychiatric disorders (Bridge et al., 2007), underscoring the need to account for developmental stage when considering the effects of alcohol on the brain and identifying neuroscience‐informed treatment targets across the lifespan.

Here, we conducted an exploratory study that aimed to address several gaps in the literature. First, we characterized the associations between neurometabolite levels and age in a sample of adolescents with moderate‐to‐high alcohol use. While the relationship between neurometabolite levels and age has been increasingly studied across the lifespan, particularly early development and older adulthood, adolescence remains understudied despite the significance of this developmental stage (Kreis et al., 1993; Pouwels et al., 1999). Second, we examined the associations between alcohol use and neurometabolite levels during adolescence to understand whether these effects align with or diverge from the predominantly adult literature. We applied 3T 1H‐MRS in 84 participants aged 17–22 with moderate‐to‐high alcohol use to investigate (1) age‐related differences in neurometabolites in the dorsal anterior cingulate cortex (dACC) and (2) associations between alcohol use and dACC neurometabolite levels. We selected dACC as our region of interest as it continues to develop late into adolescence, it plays a role in higher‐order cognitive functions, and it has been shown to be impacted by binge drinking in youth (Kalivas & Volkow, 2005; Ordaz et al., 2013; Squeglia et al., 2014). Additionally, we chose to assess all metabolites commonly quantified from 1H data, each of which has been linked to key neural functions: tNAA, tCho, GABA+, mI, Glx, and tCr. We additionally investigated the ratio between Glx and GABA+, as this ratio may be related to the excitatory/inhibitory balance in the brain, which is thought to be shifting toward inhibition during adolescence (Larsen & Luna, 2018). Findings from this study will enhance our understanding of how these neurometabolites develop through adolescence, as well as how alcohol use may affect them.

## MATERIALS AND METHODS

### Participants

This is a secondary data analysis of two completed double‐blind, placebo‐controlled, crossover clinical trials assessing the neural effects of cannabidiol (NCT05317546) or N‐acetylcysteine (NCT03238300) in non‐treatment‐seeking adolescents who use alcohol (Kirkland et al., 2023; Kirkland, Browning, et al., 2025b). The total sample across both studies was 84 participants from 17 to 22 years old (mean age = 19.5 years old, 67% female). Inclusion and exclusion criteria are briefly described here; for full inclusion and exclusion criteria for both studies see Table S1 and see Table S2 for sample demographics for each study. For the cannabidiol study, participants (*n* = 34; 17–22 years old) all met criteria for AUD within the past year, had at least one AUD symptom in the past 30 days (excluding craving), and had consumed alcohol in the past 2 weeks prior to screening. Participants in the cannabidiol study completed one scan session with placebo and one with cannabidiol, and only data from the placebo condition were used for this analysis. No carry‐over effects were found between scanning sessions in this study, suggestive of a sufficiently long washout period to clear cannabidiol before receiving placebo (Kirkland, Browning, et al., 2025b). For the N‐acetylcysteine study, participants included adolescents (*n* = 50; 17–19 years old) who reported alcohol use, including heavy alcohol use, but did not necessarily meet criteria for AUD (*n* = 30 with AUD; *n* = 20 without AUD) (26). Heavy alcohol use was defined as at least four drinking occasions with at least three drinks per occasion over 90 days prior to participating in the study (Squeglia et al., 2015). Participants in the N‐acetylcysteine study completed a baseline scan prior to being randomized to N‐acetylcysteine or placebo, and only baseline data were used for this analysis.

Exclusion criteria for both studies included as follows: (1) history of a significant or acutely unstable medical, neurological, psychiatric, or substance use problems (other than alcohol use) as assessed with the Mini‐International Neuropsychiatric Interview; (2) history of a neurodevelopmental condition that could impact brain development; (3) currently pregnant, trying to become pregnant, or breastfeeding; (4) positive urine toxicology screen for narcotics, amphetamines, sedatives, hypnotics, or opiates at screening; (5) history of treatment or treatment‐seeking for alcohol use; (6) a score of 10 or higher on the Clinical Institute Withdrawal Assessment for Alcohol; (7) acute drunkenness or consumption of alcohol within 12 h of visit (blood alcohol level of 0.00); and (8) MRI contraindications (e.g., braces, claustrophobia, irremovable metal implants, or piercings). For participants under the age of 18, parental consent and participant assent were obtained prior to collecting data. All participants were recruited from the community using mixed‐methods, and they were financially compensated for their participation. All experimental procedures were approved by the Medical University of South Carolina Institutional Review Board.

### Proton magnetic resonance spectroscopy (1H‐MRS) data acquisition

**FIGURE 1:**
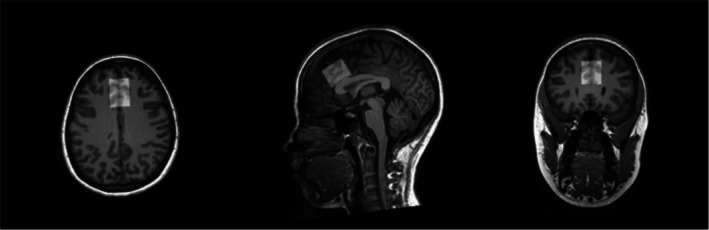
Example of typical voxel placement in dACC on a T1 image.

Both studies used the same 1H‐MRS protocols. All scans were performed on the same Siemens 3.0T Prismafit MR scanner with an actively shielded magnet and high‐performance gradients (80 mT/m, 200 T/m‐sec) using a 32‐channel head coil. High‐resolution structural scans were acquired using a magnetization prepared rapid gradient echo (MPRAGE) sequence to allow for later registration to a predefined region of interest (ROI), the dorsal anterior cingulate cortex (dACC) (scan parameters: TR/TE = 2250/4.18 ms; flip angle = 9°; field of view = 256 mm^2^; voxel size = 1 mm^2^; 176 contiguous 1‐mm‐thick slices). The 1H‐MRS protocol used was based on previously published studies (Mullins et al., 2008; Prisciandaro et al., 2020). The dACC voxel was placed on midsagittal T1‐weighted images (Figure 1), anterior to the genu of the corpus callosum, with the ventral edge of the voxel aligned with the dorsal edge of the genu (Öngür et al., 2008), with a voxel size of (30 × 25 × 25) mm^3^ (Frye et al., 2016; Öngür et al., 2008). Following FAST(EST)MAP shimming (Gruetter & Tkáč, 2000), single‐voxel water‐suppressed 1H‐MRS spectra were acquired (water suppression bandwidth: 50 Hz for Glx or 100 Hz for GABA+, spectral bandwidth 2000 Hz, 1024 spectral points). 1H‐MRS spectra were acquired with the following sequences: (1) Point Resolved Spectroscopy (PRESS) sequence: TR = 2000 ms; TE = 40 ms; number of averages = 256; (2) WIP MEGA‐PRESS sequence for GABA+: Edit‐ON(OFF) = 1.90 (7.46) ppm; TR = 2000 ms, TE = 68 ms; number of averages for condition = 160 (total of 320 averages) (Figures S1 and S2). Unsuppressed water spectra were co‐acquired and scaled for partial volume effects and relaxation and used as a concentration reference. Six saturation bands (41‐mm thickness) were placed for outer volume suppression at a distance of 0.8 cm from each voxel face.

### Quantification of metabolite levels and data quality criteria

Osprey software was used to quantify levels of metabolites from 1H‐MRS data (Oeltzschner et al., 2020). Standard parameters were used for PRESS processing (metabolite fit range: 0.2–4.2 ppm; water fit range: 2.0–7.4 ppm; knot spacing = 0.4), and parameters were optimized for GABA+ processing (metabolite fit range: 0.5–4.0 ppm; water fit range: 2.0–7.4 ppm; knot spacing = 0.55; MM09 hard modeling) (Zöllner et al., 2022). Segmentation of T1‐weighted images was done using SPM12 (Friston et al., 1994) to quantify voxel tissue composition (fraction of gray matter [GM], white matter [WM], and cerebrospinal fluid). All metabolite levels are expressed in fully tissue‐and‐relaxation‐corrected molal concentrations (mol/kg) (Gasparovic et al., 2006).

Data signal‐to‐noise ratio (ratio between amplitude of Cr peak and standard deviation of detrended noise), linewidth for Cr (full‐width half‐maximum [FWHM] of single‐Lorentzian fit to Cr peak), and linewidth for water (H_2_O; FWHM of single‐Lorentzian fit to H2O reference peak) were used as quality criteria. Individual cases were excluded from analyses if the Cr SNR was >3 standard deviations from the group mean or if the linewidth was >11 Hz.

### Alcohol use data

Alcohol and other substance use (e.g., cannabis use and nicotine use) data were self‐reported by participants using the modified Timeline Follow‐Back (TLFB) survey (Sobell & Sobell, 1992) with a reporting window over the past 60 days from their screening visit. Of note, while the N‐acetylcysteine study collected 90 days of TLFB data at screening, we only report 60 days here to match the cannabidiol study procedures. The following substance use variables were calculated and used in analyses as variables of interest and covariates: (1) total number of drinking days, (2) total number of standard drinks consumed, (3) total number of binge drinking days (4+ drinks for females, 5+ drinks for males per day), (4) maximum number of drinks consumed on a single drinking day, (5) average number of drinks per drinking day (DPDD), (6) nicotine use days, and (7) cannabis use days (summed over various methods of use for nicotine and cannabis; see Figure S3 for correlations between variables). To capture lifetime alcohol use, participants were asked a question adapted from the PhenX Toolkit “Questions for Substance Use History Form” which reads: “I know this might not be easy to remember, but in your lifetime, how many times have you consumed alcohol? (# of days)” (PhenX Toolkit, 2026). A worksheet was used to estimate yearly alcohol use occasions from the age of first initiation to current year.

### Statistical analysis

All analyses were conducted in R Statistical Software (R Core Team, 2024). To investigate associations between metabolites and age (Aim 1), we used linear regression. To test for nonlinear age‐related associations, we used generalized additive models (GAM) and Akaike's information criterion (AIC) to compare the model with the nonlinear (smooth) age term to the model with the linear age term (Akaike, 1974; Wood, 2011). In all models, a covariate was included to control for individual differences in tissue composition of the dACC voxel, the ratio of gray matter (GM) to brain matter (BM) in the voxel (GM/BM). To investigate the effect of alcohol use on metabolites (Aim 2), we used linear regression with covariates for GM/BM and age. Inclusion of other covariates was tested using the likelihood‐ratio test (Hothorn et al., 2024). Covariates tested included sex, number of cannabis use days, and number of nicotine use days to ensure that any findings were specific to alcohol use rather than general substance use. Covariates were included in the model if they made a significant difference to the model. Due to the exploratory nature of this study, *p*‐values reported were not corrected for multiple comparisons (Rubin, 2024).

## RESULTS

### Participants

Eighty‐four participants were included ranging from 17 to 22 years of age (mean age = 19.54, SD = 1.32). Sixty‐seven percent of the recruited sample reported their sex as female, and 90% of the sample reported their race as white. Seventy‐six percent of the overall sample met criteria for AUD in the past year, with 45% meeting criteria for mild AUD, 19% meeting criteria for moderate AUD, and 12% meeting criteria for severe AUD. See Table 1 for complete sample characteristics.

**TABLE 1:** Sample characteristics.

	*N* = 84 total
Age, range	17–22
Mean (SD)	19.54 (1.32)
Sex, *n* (%)^a^	
Female	56 (67%)
Male	28 (33%)
Race, *n* (%)	
Asian	3 (4%)
Black/African American	5 (6%)
White	76 (90%)
Ethnicity, *n* (%)	
Hispanic/Latino	2 (2%)
Not Hispanic/Latino	82 (98%)
Grade, *n* (%)	
10th grade	2 (2%)
11th grade	8 (10%)
12th grade/GED equivalent	33 (40%)
Some college	34 (40%)
Bachelor's Degree	7 (8%)
Mental health disorders^b^, *n* (%)	
Attention deficit hyperactivity disorder	13 (15%)
Obsessive‐compulsive disorder	2 (2%)
Major depressive episode	4 (4%)
Generalized anxiety disorder	8 (9%)
Social anxiety disorder	3 (3%)
Panic disorder	4 (4%)
Alcohol use disorder	64 (76%)
Mild	38 (45%)
Moderate	16 (19%)
Severe	10 (12%)
Cannabis use disorder	32 (38%)
Mild	14 (17%)
Moderate	12 (14%)
Severe	6 (7%)
Alcohol use^c^	
Participants with alcohol use in past 60 days, *n* (%)	84 (100%)
Number of drinking days, mean (SD)	14.49 (6.71)
Total number of standard drinks, mean (SD)	68.49 (46.58)
Number of binge drinking days, mean (SD)	8.29 (6.41)
Maximum number of drinks in 1 day, mean (SD)	9.38 (5.50)
Number of standard drinks per drinking day, mean (SD)	4.67 (2.04)
Number of lifetime drinks, mean (SD)	234.21 (295.39)
Age of first full drink	15.55 (1.67)
Past 24 h self‐reported alcohol use	13 (15%)
Past 12 h self‐reported alcohol use	0 (0%)
Other substance use^c^	
Participants with cannabis use in past 60 days	54 (64%)
Number of cannabis use days, mean (SD)	10.32 (17.11)
Urine tox screen positive for THC	17 (20%)
Participants with nicotine^d^ use in past 60 days	48 (57%)
Number of nicotine use days, mean (SD)	17.95 (23.30)

^a^ Percents are rounded to the nearest whole number.

^b^ MINI = Mini‐International Neuropsychiatric Interview; Current diagnosis was used ensuring that all diagnoses reflected participants' conditions at the time of assessment. Only diagnoses assessed between both samples were reported.

^c^ Based on 60‐day Timeline Follow‐Back (TLFB). Number of substance use days are only quantified using participants that use that substance.

^d^ Any nicotine use, including tobacco and e‐cigarettes.

### Aim 1: Age‐related associations with metabolite levels

We characterized age‐related differences in tNAA, tCho, GABA+, mI, Glx, and tCr, covarying for GM/BM in the dACC voxel. We additionally created a variable of the ratio between Glx and GABA+ to directly investigate age‐related differences in the Glx/GABA+ balance or excitatory/inhibitory balance. For all models, GAM models did not outperform linear regression models by more than 2 AIC units (Tables S3 and S4) so linear regression models were used for all models going forward for simplicity and interpretability.

**FIGURE 2:**
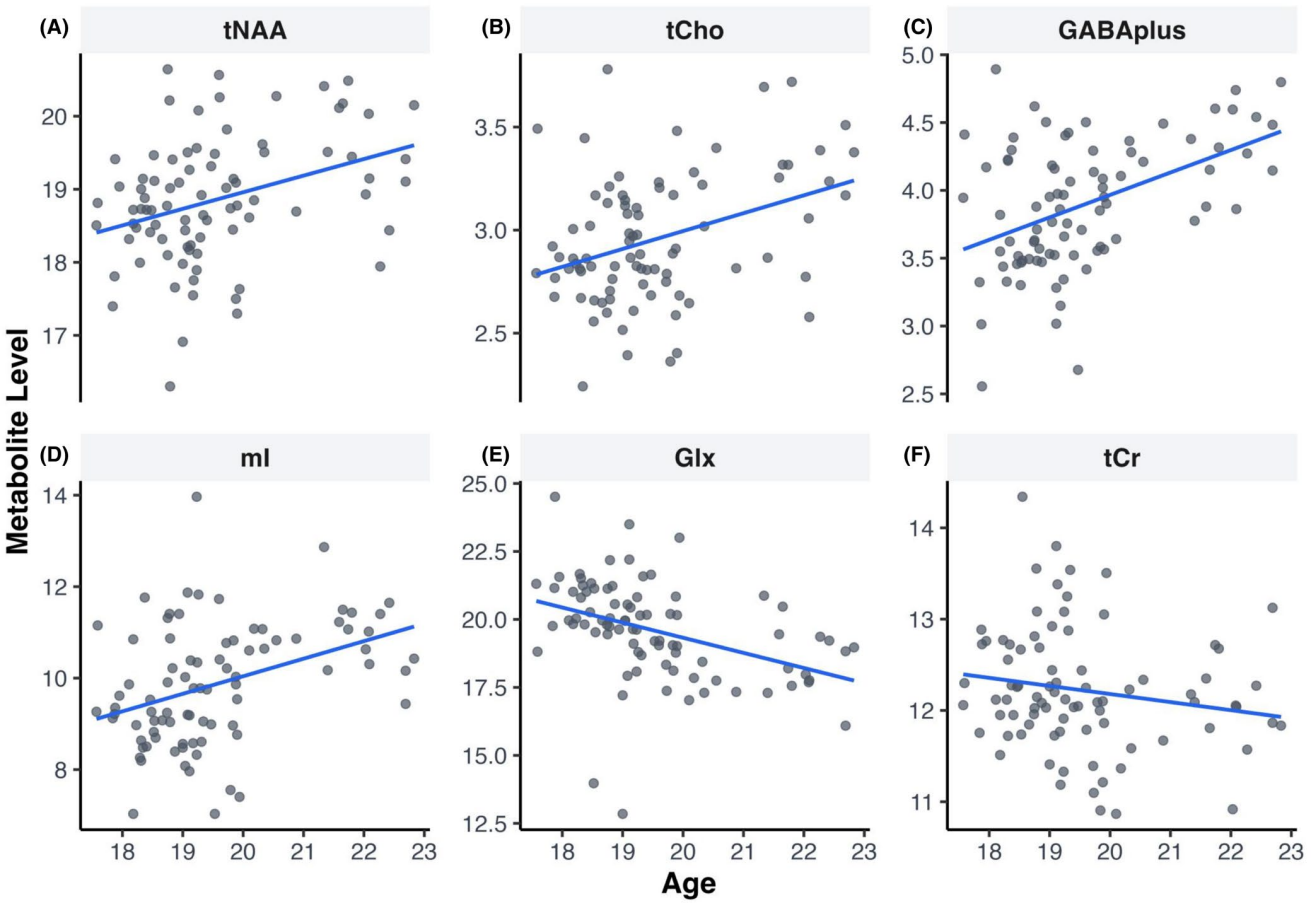
Association between metabolites and age with linear model fits. Scatter plots shown correlation between age and (A) total N‐acetylaspartate‐containing compounds (tNAA). (B) Total choline‐containing compounds (tCho). (C) GABA + macromolecules (GABA+). (D) *myo‐*Inositol (mI). (E) Glutamate + glutamine (Glx). (F) Total creatine‐containing compounds (tCr). (G) Glx/GABA+ ratio (Glx/GABA+).

Results showed that older adolescents had higher levels of tNAA (standardized coefficient *β* = 0.35, *p* = 0.001), tCho (*β* = 0.35, *p* < 0.001), GABA+ (*β* = 0.45, *p* < 0.001), and mI (*β* = 0.38, *p* < 0.001) (Figure 2; Table 2). Results showed that older adolescents also had lower levels of Glx (*β* = −0.40, *p* < 0.001) as well as a lower Glx/GABA+ ratio (*β* = −0.48, *p* < 0.001). Finally, results also showed no association with age for tCr levels (*β* = −0.17, *p* = 0.13).

**TABLE 2:** Linear model output for associations between neurometabolites and age.

Metabolite	Predictor	*β*	*β*, 95% CI [LL, UL]	*t*	*p*
tNAA	**Age**	0.35	[0.15, 0.56]	3.39	0.0011**
	GM/BM	−0.11	[−0.32, 0.09]	−1.09	0.28
tCho	**Age**	0.35	[0.15, 0.56]	3.44	<0.001**
	GM/BM	0.15	[−0.05, 0.36]	1.48	0.14
GABA+	**Age**	0.45	[0.25, 0.65]	4.56	<0.001**
	GM/BM	0.08	[−0.11, 0.28]	0.85	0.40
mI	**Age**	0.38	[0.18, 0.58]	3.70	<0.001**
	GM/BM	0.07	[−0.13, 0.28]	0.73	0.47
Glx	**Age**	−0.40	[−0.60, −0.20]	−3.94	<0.001**
	GM/BM	0.00	[−0.20, 0.21]	0.034	0.97
tCr	Age	−0.17	[−0.39, 0.05]	−1.55	0.13
	GM/BM	−0.10	[−0.31, 0.12]	−0.88	0.38
Glx/GABA+	Age	−0.48	[−0.67, −0.29]	−4.92	<0.001**
	GM/BM	−0.05	[−0.24, 0.15]	−0.49	0.62

*Note*: β indicates standardized coefficients. LL and UL indicate the lower and upper limits of a confidence interval, respectively. Bolded predictor variables represent *p* < 0.05. * indicates *p* < 0.05. ** indicates *p* < 0.01.

### Aim 2: Alcohol‐related associations with metabolite levels

We then assessed how variation in alcohol use was associated with metabolite levels. For all base models, we included GM/BM and age as covariates. We additionally used the likelihood‐ratio test to test whether including sex, number of cannabis use days, or number of nicotine use days would improve model fit. For all models, we found that these covariates did not improve fit above the base model, and thus, these covariates were not included going forward (Table S5). Additionally, we found no associations between age and all our alcohol variables (*p*‐values >0.05).

**FIGURE 3:**
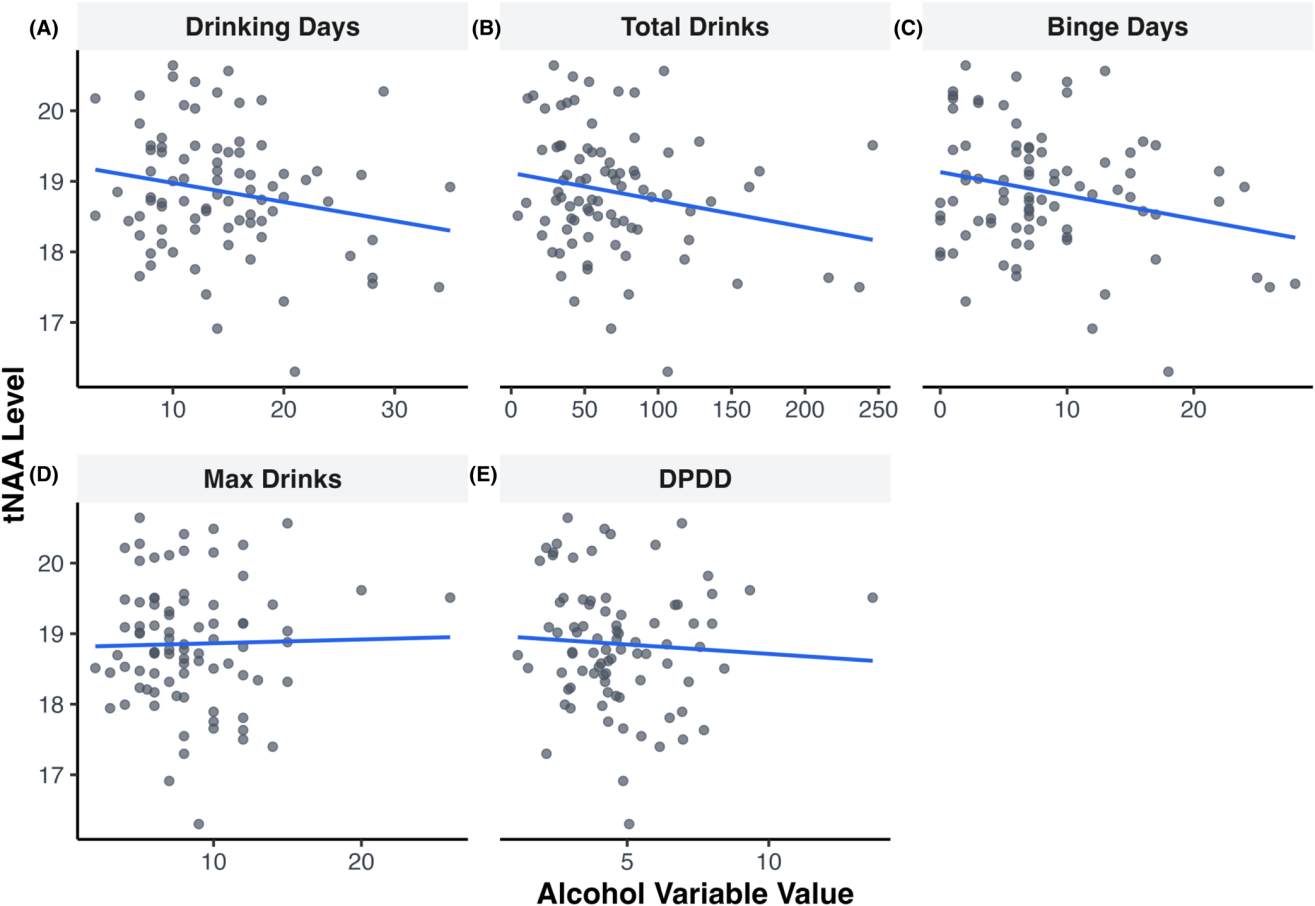
Association between alcohol (past 60 days) and total N‐acetylaspartate‐containing compounds (tNAA) with linear model fits. (A) Days spent drinking alcohol. (B) Total drinks consumed. (C) Maximum number of drinks consumed in one drinking episode. (D) Drinks per drinking day. (E) Binge drinking episodes.

Results showed that levels of tNAA were associated with self‐reported alcohol use (Table 3). Specifically, there were lower levels of tNAA with higher numbers of drinking days (*β* = −0.26, *p* = 0.012), higher numbers of total drinks consumed (*β* = −0.21, *p* = 0.043), and higher numbers of binge drinking days (*β* = −0.22, *p* = 0.037) (Figure 3). No other metabolites were associated with alcohol use (Table S6). Additionally, we found no associations between any neurometabolites and self‐reported estimates of the number of lifetime drinking days (Table S7).

**TABLE 3:** Linear model output for associations between tNAA and alcohol variables.

Predictors	*β*	*β*, 95% CI [LL, UL]	*t*	*p*
*Model: tNAA ~ Drinking Days + Age + GM/BM*				
**Drinking days**	−0.26	[−0.46, −0.06]	−2.56	0.012*
**Age**	0.38	[0.18, 0.58]	3.76	<0.001**
GM/BM	−0.14	[−0.34, 0.06]	−1.39	0.17
*Model: tNAA ~ Total Drinks + Age + GM/BM*				
**Total drinks**	−0.21	[−0.41, −0.01]	−2.06	0.043*
**Age**	0.34	[0.14, 0.55]	3.39	0.0012**
GM/BM	−0.14	[−0.34, 0.07]	−1.35	0.18
*Model: tNAA ~ Binge Days + Age + GM/BM*				
**Binge days**	−0.22	[−0.43, −0.01]	−2.12	0.037*
**Age**	0.32	[0.12, 0.52]	3.11	0.0026**
GM/BM	−0.14	[−0.35, 0.06]	−1.39	0.17
*Model: tNAA ~ Max Drinks + Age + GM/BM*				
Max Drinks	0.05	[−0.16, 0.26]	0.47	0.64
**Age**	0.36	[0.15, 0.56]	3.40	0.0011**
GM/BM	−0.11	[−0.32, 0.09]	−1.10	0.26
*Model: tNAA ~ DPDD + Age + GM/BM*				
DPDD	−0.01	[−0.22, 0.20]	−0.07	0.95
**Age**	0.35	[0.14, 0.56]	3.32	0.0014**
GM/BM	−0.11	[−0.32, 0.10]	−1.08	0.28

*Note*: Over the past 60 days, “Drinking Days” refers to the number of days alcohol was consumed. “Total Drinks” refers to the total number of drinks consumed. “Binge Days” refers to the number of binge drinking episodes. “Max Drinks” refers to the maximum number of drinks consumed in one drinking episode. “DPDD” refers to the average number of drinks per drinking day. *β* indicates standardized coefficients. LL and UL indicate the lower and upper limits of a confidence interval, respectively. Bolded predictor variables represent *p* < 0.05. * indicates *p* < 0.05. ** indicates *p* < 0.01.

## DISCUSSION

In this study, we provide novel evidence of age‐related differences in neurometabolites within the dACC during late adolescence in a sample of adolescents with moderate‐to‐high alcohol use, as well as the associations between alcohol use and neurometabolite levels. We identified that older adolescents had higher levels of neurometabolites involved in neuronal health (tNAA), lipid membrane synthesis and glial markers (tCho, mI), and inhibition (GABA+), alongside lower levels of excitatory neurotransmission (Glx). The excitatory/inhibitory ratio (Glx/GABA+) is lower in older adolescents. In contrast, a marker of energetic/metabolic activity (tCr) did not vary with age. Notably, we found that more recent alcohol use was associated with lower tNAA levels, in direct opposition to the positive association between age and tNAA that we observed. Interestingly, we did not find any significant associations between alcohol use and the other metabolites we investigated, or any associations between lifetime drinking and any neurometabolites. Furthermore, cannabis and nicotine use did not show any significant effects on neurometabolites over and above the effects of alcohol use. These findings offer new insights into the age‐related variation in dACC neurometabolite levels during adolescence and highlight tNAA as a potential early marker of neuronal damage linked to alcohol use.

### Neurometabolite levels during adolescence

Although our sample consisted of adolescents with moderate‐to‐high alcohol use, our results contribute evidence toward characterizing neurometabolite levels during adolescence in the dACC, while also replicating and extending prior work. Our findings of higher levels of inhibitory neurometabolites (GABA+), and lower levels of excitatory neurometabolites (Glx) and the ratio of Glx/GABA+ in older adolescents aligns with findings from preclinical animal model literature suggestive of a shift in the balance between excitatory and inhibitory neurotransmission in executive brain regions, such as dACC, which could support cognitive and socioemotional development (Larsen & Luna, 2018). Our observed negative association between age and lower dACC Glx may reflect known developmental pruning of primarily excitatory glutamatergic synapses (Huttenlocher, 1990; Perica et al., 2022; Petanjek et al., 2011). This finding is consistent with some recent human 1H‐MRS studies of normative adolescent neurodevelopment (Perica et al., 2022; Shimizu et al., 2017; Widegren et al., 2024), although this pattern has not been observed across all prior work (Ghisleni et al., 2015; Gleich et al., 2015; Silveri et al., 2013). Additionally, we observed a positive relationship between dACC GABA+ levels and age, which may reflect ongoing maturation of inhibitory neurotransmission in frontal and association cortex across adolescence (Larsen & Luna, 2018). This finding is consistent with prior work reporting higher dACC GABA+ levels in older adolescents (18–24 years old) relative to younger adolescents (Silveri et al., 2013), but again, associations between GABA and age in dACC have not been observed across all studies (Perica et al., 2022; Widegren et al., 2024). Finally, our findings of a negative association between Glx/GABA+ ratio and age are in line with preclinical findings from the broader literature that suggest shifts in the balance between excitation and inhibition during adolescence wherein there are increases in inhibition relative to excitation (Larsen & Luna, 2018). However, this contradicts the only other study to examine this that found no associations between age and Glu/GABA ratio in dACC (Perica et al., 2022). This could be due to a variety of methodological differences from sample size and composition (non‐alcohol‐using youth in Perica et al., 2022 vs. alcohol‐using in the present manuscript) to acquisition to processing pipeline. These issues underscore the need for further replication, as well as larger, longitudinal studies to clarify these within‐person trajectories. These large‐sample prospective studies are also needed to clarify baseline trajectories of Glx, GABA, and other metabolites in non‐alcohol‐using youth in order to better distinguish normative developmental trajectories from alcohol‐related effects.

Far less is known about the patterns of the other neurometabolites in the dACC during adolescence. In our sample, we found a positive association between age and tNAA levels. NAA is abundant in neurons, but despite that, its specific function remains obscure (Baslow, 2000). NAA has been thought to be a marker of neuronal, axonal, and dendritic viability, as reduced NAA levels are consistently observed in neurodegenerative and neuropsychiatric disorders where neuronal loss or dysfunction is present (Barker, 2001). Therefore, it is possible that age‐related increases in tNAA during adolescence may reflect the development of synapses, dendrites, and neurons (Kato et al., 1997; Spear, 2013). Consistent with our findings in 17–22 year olds, another study found age‐related increases in dACC NAA in a sample of 234 9–12 year olds (Baron Nelson et al., 2019). However, two smaller studies found no differences between younger adolescent and older adolescent/adult age groups (Shimizu et al., 2017; Silveri et al., 2013); however, the studies may have been underpowered and used different methodological approaches that could limit the ability to detect subtle variation. Our findings add to this limited literature by providing preliminary evidence to inform future longitudinal hypotheses examining the maturation and neurodevelopmental role of tNAA during adolescence.

Similarly, relatively little research has investigated tCho or mI in dACC during adolescence. Here, we find a positive association between age and tCho and mI. Both Cho and mI are thought to be markers of processes related to lipid membranes, in addition to mI being thought of as a marker for glial cells (Brand et al., 1994; Rae, 2014). Increases during the adolescent period may reflect changes in lipid membranes, such as known developmental increases in myelination of axons during adolescence, or maturation of glia (Baum et al., 2022; Rae, 2014). However, as with NAA, the function of both metabolites, especially their roles in neurodevelopment, is not well understood. To our knowledge, only two studies have reported on tCho through adolescence with mixed results, where one study found higher levels of tCho in a group of 9–12 year olds with age (Baron Nelson et al., 2019), and another study reported no difference between two groups of younger and older adolescents in tCho/Cr or mI/Cr (Silveri et al., 2013).

Finally, we find no age‐related associations in tCr through adolescence. To our knowledge, only one other study has reported age effects for tCr in dACC, and this study found higher tCr levels with age in a group of 9–12 year olds (Baron Nelson et al., 2019). The limited literature on tCr likely stems from its common use as a reference metabolite for normalization, rather than as a primary focus of study. However, tCr plays a critical role in energetic and metabolic processes in the brain and more research is needed to understand the role it may be playing during development (Rae, 2014). Our use of water‐referencing in this study allows us to study tCr directly and contributes novel information about tCr during later adolescence. Furthermore, referencing to Cr as a general practice presents a number of issues, such as increasing the variability and noise when creating ratios of metabolites to tCr (Li et al., 2003; Rae, 2014; Wilson et al., 2019). Therefore, our use of water‐referenced values contributes valuable information to the literature about all our reported neurometabolites.

### Alcohol use and Neurometabolite levels

Our findings also provide novel information about the association between alcohol use and neurometabolite levels during adolescence, specifically that more drinking days, binge drinking, and total number of drinks consumed in the past 60 days are associated with lower tNAA levels. This finding is similar to those seen with tNAA in a study of 18–24 year olds that noted a negative association between lower tNAA levels in dACC and binge drinking (Silveri et al., 2014), as well as the extant adult literature (Kirkland et al., 2022). Lower levels of tNAA may represent neuronal damage as a result of alcohol use, as lower NAA levels are often seen in a variety of neurodegenerative disorders (Barker, 2001). Furthermore, it is known that alcohol has neurodegenerative effects in youth, including decreased gray matter volume and cortical thickness (Lees et al., 2021). However, given the cross‐sectional nature of this study and much of the current literature, the interpretation of lower tNAA levels associated with alcohol remains speculative. These lower levels may represent neuronal damage resulting from alcohol use or they could represent maturational delay, as we found a positive relationship between tNAA and age. Findings could also be a result of a third variable correlated with alcohol use, but longitudinal studies are needed to truly answer this question. Given the remarkable consistency of lower tNAA levels associated with more alcohol use in our study, NAA may have value as a potential early biomarker or treatment target warranting further investigation.

The lack of alcohol‐related findings in the other neurometabolites of interest within the dACC aligns with the majority of the extant literature in adolescents and adults (Kirkland et al., 2022). A prior analysis of a subset of the current study's sample did not detect any neurometabolite differences between an alcohol‐using group and non‐alcohol‐using group, including no differences in tNAA, possibly due to limited sample size, restricted age range, and alcohol use severity (Kirkland et al., 2024). However, in contrast to our findings, two studies did report alcohol‐related associations with GABA (Marinkovic et al., 2022; Silveri et al., 2014). These different findings could be a result of the difficulty in reliably measuring GABA due to the dynamic nature of GABA synthesis or its low concentration in the brain (Meyerhoff, 2014) or the slightly older average age of those samples, suggesting that broader neurometabolic effects may emerge later in development. It is possible that the highly plastic nature of the younger adolescent brain may allow for greater recovery potential (Larsen & Luna, 2018), thus resulting in fewer long‐lasting neural changes following alcohol use.

Notably, in contrast to past 60‐day alcohol use, we did not find associations between estimated lifetime drinking days and any of our reported neurometabolites. Therefore, it is possible that more recent drinking behavior may have a stronger effect on neurometabolite levels, or it may be that participants are better able to accurately recall alcohol use on a shorter and more recent timeframe (Kirkland, Ferguson, et al., 2025a). Furthermore, although we systematically tested both cannabis use days and nicotine use days, neither emerged as significantly associated with neurometabolite levels beyond the impact of alcohol use. A systematic review of alcohol and cannabis use during adolescence on structural and functional neurodevelopment found more pronounced neural effects relating to alcohol use at lower levels than to cannabis use (Lees et al., 2021). However, neural effects of cannabis use and nicotine use have certainly been noted (Colyer‐Patel et al., 2023; Lichenstein et al., 2022). Our lack of findings here may have been due to being underpowered to detect these effects, as participants were recruited specifically based on their heavy alcohol use and not all of them reported co‐occurring cannabis or nicotine use.

### Strengths and limitations

This study has several notable strengths. First, this study's inclusion of multiple neurometabolites provides a more comprehensive view of neurochemical development during adolescence. Our use of water‐referencing in our 1H‐MRS data to quantify neurometabolites overcomes some of the limitations caused by normalizing to creatine, as is commonly done across the 1H‐MRS literature. Furthermore, a key strength of this study is the use of a relatively large and developmentally younger sample compared with many previous 1H‐MRS studies both of development and of alcohol use, enhancing the generalizability of our findings to alcohol exposure in youth broadly. While this sample used alcohol, none of the participants were treatment‐seeking for AUD and the majority either had no AUD (24%) or mild AUD (45%), making this a more representative sample of young people who use alcohol. Importantly, our use of an adolescent sample allowed us to highlight NAA as a specific and early marker of alcohol's effects on the brain, which differs from more widespread findings in the adult literature and highlights the need for further research into NAA and its role in neurodevelopment.

However, several limitations should be noted. First, we present cross‐sectional data, which limits our ability to draw causal inferences or assess within‐person change. Second, we only present data from one brain region which, although common in 1H‐MRS studies, limits our ability to understand impacts on other regions or broader networks. Furthermore, there are some methodological challenges that remain when quantifying metabolites with 1H‐MRS, including difficulty separating the overlapping signals of glutamate and glutamine (thus the compound measure Glx), difficulty separating GABA from overlapping macromolecule signal (thus GABA+), as well as relatively large voxel size (Bell et al., 2021; Choi et al., 2006; Ramadan et al., 2013). Longitudinal studies that include a variety of brain regions are needed to determine whether alcohol use leads to widespread prospective alterations in neurometabolite trajectories over the course of development. Third, although alcohol use is normative and common among adolescents in the United States (Substance Abuse and Mental Health Services Administration, 2024), our sample included only adolescents that reported moderate‐to‐heavy alcohol use. Future studies should aim to build on our findings with longitudinal data from non‐alcohol‐using samples to help further clarify baseline developmental trajectories of these critical neurometabolites. Fourth, our overall sample is predominantly female (67%), which presents some concerns to both generalizability, as well as potential confounds, such as fluctuating hormonal differences that can impact measured metabolite levels (Hjelmervik et al., 2018). However, substance use literature has historically overrepresented men, and thus, predominantly female samples provide valuable novel information as well. Finally, although our sample size is larger than in many prior studies, replication in larger and more diverse samples will be needed to confirm and extend these findings to the broader population.

## CONCLUSIONS

Our exploratory study provides valuable information about neurometabolite variation in adolescence, in addition to associations with alcohol use. We report a variety of age‐related differences in several neurometabolites, with older adolescents having higher levels of neurometabolites critical for processes involved in neuronal health and integrity (tNAA), lipid membrane synthesis and glial function (tCho, mI), and inhibitory neurotransmission (GABA+), concurrent with lower levels of excitatory neurotransmission (Glx) and a lower ratio between excitatory and inhibitory neurotransmission (Glx/GABA+). Additionally, we found that a marker of metabolic activity (tCr) was not associated with age. Furthermore, alcohol use during adolescence was associated with lower levels of tNAA but not other metabolites, which was specific to more recent alcohol use rather than lifetime use. The contrast between the age‐related increase in tNAA and alcohol‐related decrease in tNAA suggests that alcohol use may be associated with patterns that differ from typical neurodevelopmental patterns of tNAA. Together, these findings point to tNAA as a promising target for future research on early markers of alcohol‐related neural effects as well as novel treatment targets for youth alcohol use disorders.

## CONFLICT OF INTEREST STATEMENT

The authors report no financial disclosures or potential conflicts of interest.

## SOURCES OF SUPPORT

LS: NIAAA R21AA030114, NIAAA K23AA025399, NIAAA K24AA031052. AK: NIAAA K01AA031745. MP: NIDA T32DA007288, NIAAA L30AA032673.

## Supporting information


Figures S1–S3
Tables S1–S7

## Data Availability

The data that support the findings of this study are available from the corresponding author upon reasonable request.
